# The Panopticon—Assessing the Effect of Starvation on Prolonged Fly Activity and Place Preference

**DOI:** 10.3389/fnbeh.2021.640146

**Published:** 2021-03-25

**Authors:** Deepthi Mahishi, Tilman Triphan, Ricarda Hesse, Wolf Huetteroth

**Affiliations:** Department of Genetics, Faculty of Life Sciences, University of Leipzig, Leipzig, Germany

**Keywords:** *Drosophila*, feeding, foraging, place preference, tracking

## Abstract

Animal behaviours are demonstrably governed by sensory stimulation, previous experience and internal states like hunger. With increasing hunger, priorities shift towards foraging and feeding. During foraging, flies are known to employ efficient path integration strategies. However, general long-term activity patterns for both hungry and satiated flies in conditions of foraging remain to be better understood. Similarly, little is known about how permanent contact chemosensory stimulation affects locomotion. To address these questions, we have developed a novel, simplistic fly activity tracking setup—the Panopticon. Using a 3D-printed Petri dish inset, our assay allows recording of walking behaviour, of several flies in parallel, with all arena surfaces covered by a uniform substrate layer. We tested two constellations of providing food: (i) in single patches and (ii) omnipresent within the substrate layer. Fly tracking is done with FIJI, further assessment, analysis and presentation is done with a custom-built MATLAB analysis framework. We find that starvation history leads to a long-lasting reduction in locomotion, as well as a delayed place preference for food patches which seems to be not driven by immediate hunger motivation.

## Introduction

Flies show hunger-motivated ranging or foraging walks to find food; they also show explorative walks (or local searching behaviour) after food encounter (Dethier, 1957; Bell et al., 1985; Bell, 1990; Corrales-Carvajal et al., 2016; Kim and Dickinson, 2017; Murata et al., 2017; Hughson et al., 2018; Mahishi and Huetteroth, 2019), and much has been achieved in identifying the circuits and dynamics involved in this behaviour (Corfas et al., 2019; Lin et al., 2019; Moreira et al., 2019; Sayin et al., 2019; Seidenbecher et al., 2020; Behbahani et al., 2021). Most of these studies either used hunger-motivated behaviour to focus on the underlying navigational strategy of the flies, or they focussed on exploration–exploitation trade-offs under different motivational settings.

Interestingly, both hedonic and caloric value of the food source can influence explorative walks. The perceived sweetness after ingestion correlates to the duration and path length of explorative walking (Murata et al., 2017), and protein-sated flies venture further away from a yeast patch than protein-deprived flies (Corrales-Carvajal et al., 2016). As satiation levels drop with ongoing post-prandial energy expenditure, finding food is becoming increasingly important. An exploring fly constantly assesses palatability with its tarsal chemosensors, supported by occasional proboscis extension (Mahishi and Huetteroth, 2019). But how much does a fly explore when nutritional homeostasis can be achieved anywhere?

What are the locomotion dynamics independent of food search? To overcome distorting foraging locomotion, driven by constantly changing hunger levels, we provide an arena with omnipresent food. Although these previous studies imply foraging-independent explorative walking in sated flies (Bell et al., 1985; Bell, 1990; Murata et al., 2017), no study exists to our knowledge that studied prolonged intrinsic walking behaviour on a homogenous food substrate, where any locomotion motivated by food-seeking can be ruled out.

It is also not well understood how palatability and satiation affect walking activity beyond the first 3–120 min after food interaction; food-related responses can exert their physiological and behavioural effects on longer timescales. Larval diet composition impacts adult food choice (Davies et al., 2018), and preference of a caloric diet over an equally palatable alternative is only established after several hours (Dus et al., 2011; Stafford et al., 2012). Similarly, a dietary imbalance between palatability and nutritional content is leading to sustained physiological changes much later (Wang et al., 2016, 2017; Musso et al., 2017; Park et al., 2017; May et al., 2019). Apart from few exceptions (Martin, 2004; Meunier et al., 2007), most existing fly locomotion studies either examine short periods at high temporal resolution (Kim and Dickinson, 2017; Murata et al., 2017; Brockmann et al., 2018; Hughson et al., 2018; Landayan et al., 2018), or sample for short recurring time windows to cover longer periods (Green, 1964a,b; Connolly, 1966a; Barwell et al., 2020). Automated circadian studies provide both temporal resolution and timespan, but focus on changes in rhythmicity (Guo et al., 2016; Pegoraro et al., 2020), and rarely include location preferences or locomotion in a nutritional context (Donelson et al., 2012; Dreyer et al., 2019).

We use uninterrupted video tracking (1 Hz) of pre-starved and pre-fed flies to compare locomotion activity and location probability for over 24 h in two conditions: (i) a foraging setting with a single food patch or (ii) with homogenous food substrate on all surfaces. Our assay comes with a data pipeline from recording to analysis utilising custom-written camera recording software, FIJI-based tracking, and MATLAB-based data analysis, various sanity check functions and visualisation.

## Materials and Methods

### Animals

**FIGURE 1:**
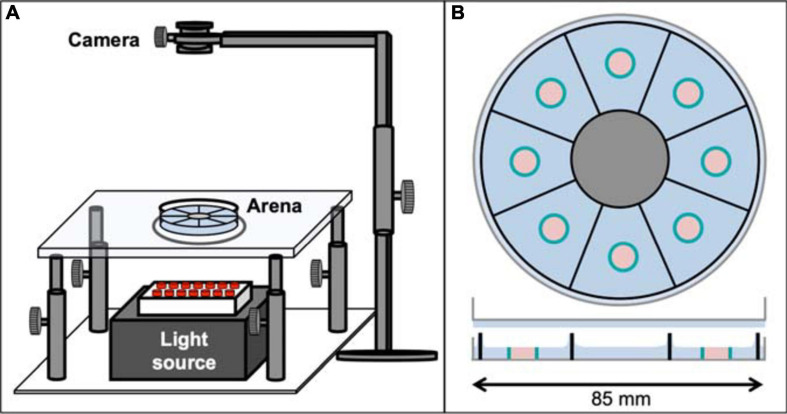
The Panopticon. **(A)** Entire recording setup for the Panopticon. The whole assay platform has an infrared light source placed in the bottom (grey box with red LEDs), on top of which is a height-adjustable transparent glass platform with a small circular plastic layer acting as diffusor. The arena itself is placed on this platform, positioned precisely in line with the light source, the diffusor and the recording camera fitted on top with a stable holder. **(B)** Top view of Panopticon, sagittal view below. The arena consists of eight visually separated sectors, as indicated by sector-dividing walls of the 3D printed inset (black), which sits in substrate (blue) inside a Petri dish lid (grey). Another Petri dish lid with a layer of substrate closes the arena. Two configurations are used: (i) food patch arena with 1% agarose as substrate (depicted), which contains individual, centrally located food patch containers (teal blue) filled with 1% agarose with 200 mM sucrose (pastel pink) or (ii) omnipresent food arena without food patches, but 1% agarose with 200 mM sucrose as substrate (not shown).

All experiments were performed with 2–5 days old male Oregon^R^ flies, maintained at the University of Leipzig at 25°C and 60% humidity on a 14:10 LD cycle (light 7:00 to 21:00) on standard fly food. Pre-starved animals were kept for 22–24 h in empty vials with added wet tissue, pre-fed flies were allowed to feed *ad libitum* on normal fly food, until placing them in the Panopticon under constant dark conditions, with infrared LED lighting (850 nm) from below (Figure 1A). The average starting time of experiments was 12:00 ± 2 h for foraging experiments or 12:30 ± 3 h for omnipresent food experiments, respectively.

### Panopticon Assay

Cold anesthetised pre-fed and pre-starved male flies were alternately placed in individual sectors of the Panopticon and then transferred onto the imaging rig (Figure 1A), which is located inside a climate chamber (not shown) to maintain constant levels of 60% humidity and a temperature of 25°C. The Panopticon consists of an opaque 3D-printed arena (.stl file; Renkforce RF1000: Material PLA white), which is inserted in the lid of a standard plastic Petri dish (85 mm, Greiner) partially filled with substrate (1% agarose or 1% agarose containing 200 mM sucrose), separating it in eight sectors (5.5 cm^2^ each) and prohibiting any sensory contact between flies (Figure 1B). During insertion of the plastic arena into still viscous substrate we assured homogenous coating of all inner walls before solidification. For foraging experiments, small Eppendorf lids were used to create individual food containers in each arena (food patches, 0.2 cm^2^). The 1% agarose with 200 mM sucrose in these food patches was levelled with the surrounding 1% agarose to avoid confounding effects of negative geotaxis on place preference (Robie et al., 2010). The Panopticon was closed with another inverted Petri dish lid with a layer of substrate to provide equal surface texture on all sides (Figure 1B). Data collection was started as soon as all flies regained walking ability (within 1 min of transfer). Images (1024 × 1024 px) were recorded with a camera (Basler acA1300-200uc) at 1 frame/s for 24 h.

### Tracking and Analysis

The recorded images were processed in batches using a custom-written, FIJI-based macro. Object information was extracted and saved as csv files. Results were then further handled using a data analysis script written in MATLAB 2018a, which provides various sanity check functions and visualisation. Some plots make use of the “shadedErrorBar” function (Campbell, 2020). Activity and place preference plots are either aligned according to start time (1 h plots) or time of day (24 h plots). To exclude movements caused by camera noise we set a minimum threshold of 2 pixels (0.1 mm) and treated the fly as stationary in this case. Regions of interest for place preference (food patches, same-sized virtual food patches) were created in MATLAB. For further details on analysis and plotting functions see Results and Code, and the Readme file available on Github^1^. Fast and efficient data handling allows for tracking and analyses of 24 h data (recorded at 1 Hz) in about 4 h on a regular desktop PC (Ryzen 3 3200G, 16 GB RAM, Intel SSD 660p 512GB).

### flyPAD

Food sip measurements were performed on the flyPAD (Itskov et al., 2014). Animals were pre-fed or pre-starved as before, anesthetised on ice and then transferred into individual flyPAD chambers with added wet tissue to allow for 24 h recordings. Data analysis took place with a customised version of the MATLAB script for the flyPAD (Itskov et al., 2014).

### Statistics

All statistical analyses were conducted using GraphPad Prism 8^®^. Mann–Whitney U-tests were performed between pre-fed and pre-starved fly values in each bin for activity plots, place preference plots, stop duration plots and flyPAD sip plots. For displacement distribution plots, values for short-distance walks (≤2 mm) and, where applicable, long-distance walks (5–10 mm) of pre-fed and pre-starved flies were binned and compared with a Mann–Whitney U-test. Horizontal lines indicate significance levels between pre-fed and pre-starved flies from a single group, curly braces represent identical significance levels for multiple groups (^∗^ = significant difference, alpha = 0.05; n.s. = not significant).

## Results

### Paradigm and Data Pipeline for Image Based Tracking of Fly Locomotion

We designed a novel, low-cost fly activity tracking setup, the Panopticon (Figure 1). Walking behaviour of eight flies can be recorded simultaneously for up to 24 h. All arena walls and the top lid are covered in 1% agarose (with or without 200 mM sucrose) to both provide equal surface texture on all sides and to maintain humidity levels and avoid desiccation, allowing continuous recordings for up to a week (data not shown). Experiments were performed in constant darkness (DD) with IR lighting (850 nm) to provide constant environmental conditions. It has been previously shown that DD is less disruptive to fly activity pattern than constant light (LL) (Green, 1964a).

**FIGURE 2:**
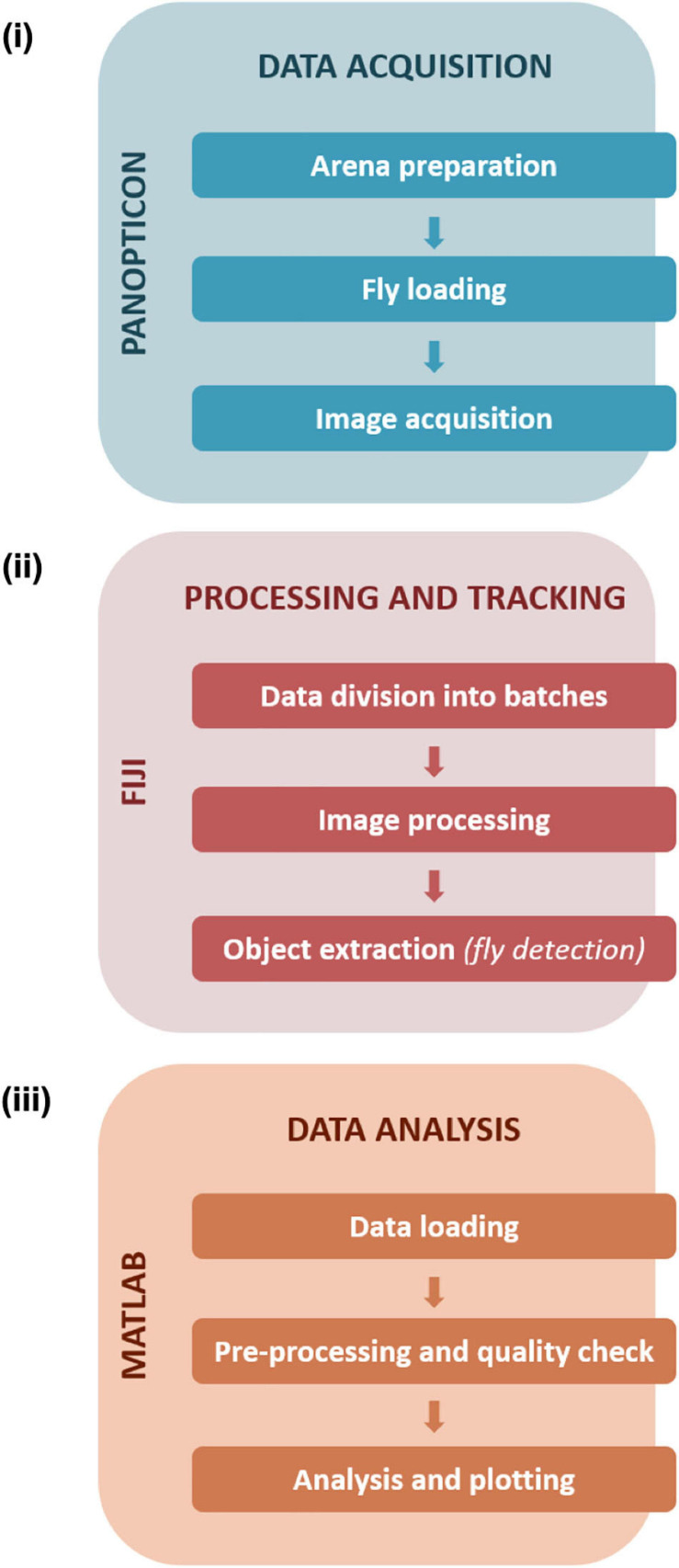
Data handling and workflow. Data acquisition **(i)** steps with the Panopticon involve preparation of the arena with substrate media, collection and loading of single flies in each of the sectors, followed by recording of fly activity. Further processing and tracking **(ii)** of recorded data (in JPEG format) is performed on FIJI to further divide collected frames into batches to facilitate efficient image processing and subsequent detection of fly positions. Data analysis **(iii)** was executed in MATLAB and consisted of pre-processing and quality checks followed by in-depth analysis and plotting of the tracked data (saved and loaded as.csv files).

We devised a complete data pipeline from recording to analysis (Figure 2). Image recording utilises custom-written camera recording software. Subsequently, the recorded images are analysed in batches using a custom-written, FIJI-based macro script. After background subtraction and pre-processing of the images objects are extracted and saved. The results are further analysed in a custom, MATLAB-based data analysis framework. Here, basic quality control and error checking functions are applied. At first, we calculated the rate of missed/failed detections, and experiments with an error rate of more than 5% were excluded from further analysis (Supplementary Figure 1A). In the second step, we did a visual inspection of the walking traces (shown with a temporal colour code) to check for obvious detection errors (Supplementary Figures 1B,C). As the last step we did a sector-wise plotting of particularly long frame-to-frame movement events that could be associated with potentially false-positive detections (Supplementary Figure 1D). After this initial quality control, derived parameters like activity patterns and location probabilities are calculated.

### Pre-starved Flies Appear Sated Within 20–30 min of Food Provision

**FIGURE 3:**
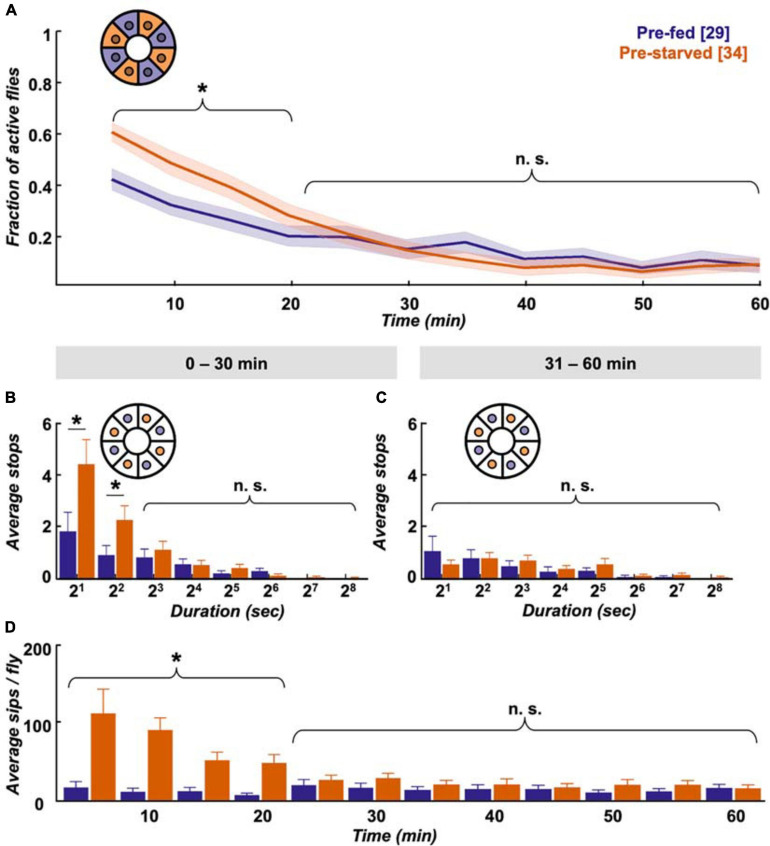
Pre-starved flies show higher activity and food intake within first 30 min. **(A)** Both pre-fed (deep purple) and pre-starved (orange) flies exhibit raised activity on the food patch assay (inset schematic) during the first 20 min, with pre-starved flies showing significantly higher activity than pre-fed flies. Both groups reach baseline levels subsequently. Data is presented as means ± SEM in 5 min bins. *N* values are given in brackets. **(B)** Initially starved flies make significantly more stops of 2–7 s duration than pre-fed flies in the first 30 min, **(C)** but not in the next 30 min on the food patch (inset schematic). **(D)** Feeding rate, as measured on the flyPAD, indicates that initially starved flies show higher initial sip rate than the pre-fed flies, which quickly decreases to a comparable sip rate. *Indicate significance levels following Mann–Whitney U-tests (alpha = 0.05), n.s. = not significant. Horizontal lines represent comparison between pre-fed and pre-starved flies from a single group. Curly braces represent identical significance levels across multiple groups. *N* values are given in brackets.

First we assessed how hunger impacts locomotion activity during food search, and within which timescale the effect subsides after provision of food. Finding a food patch is well studied, and we know from existing data that locomotion increases with starvation level (Connolly, 1966b; Knoppien et al., 2000) and changes its dynamics after food patch encounter (Dethier, 1976; Corrales-Carvajal et al., 2016; Kim and Dickinson, 2017; Murata et al., 2017). Using the Panopticon with food patches, we find that both pre-starved and pre-fed flies exhibit increased activity for about 20–30 min in their new environment, as reported before (Liu et al., 2007; Soibam et al., 2012). This initial increased activity is significantly more pronounced in pre-starved flies during the first 20 min (Figure 3A), which correlates with compensatory overeating as soon as food is available (Carvalho et al., 2005). Flies need to stop to feed, and there is a reciprocal relation between the two behaviours (Mann et al., 2013). Accordingly, there is a significantly higher number of short duration stops (2–7 s) on the food patch during the first 30 min (Figure 3B), which soon shift during the subsequent 30 min towards less frequent, longer breaks (Figure 3C). At this point, both pre-starved and pre-fed flies reach equally low levels of activity (Figure 3A). This pattern is reflected in sip numbers, as independently determined on the flyPAD, which provides a good estimate of actual food intake (Itskov et al., 2014). Pre-starved flies exhibit a significantly higher sip number as pre-fed flies at first, but sip numbers quickly reach equal baseline levels (Figure 3D), suggesting comparable satiation in both initially starved and fed flies. This is in accordance with previous studies that demonstrated reduced activity after a meal (Murphy et al., 2016).

Taken together, our data shows that during the first 20–30 min pre-starved flies exhibit increased activity, and an increased number of stops lasting between 2 and 7 s. These most likely reflect feeding bouts, which soon disappear when they presumably reach hunger motivation levels comparable to pre-fed flies during the following 30 min.

### Starvation State Affects 24 h Walking Activity and Place Preference in a Food Patch Assay

**FIGURE 4:**
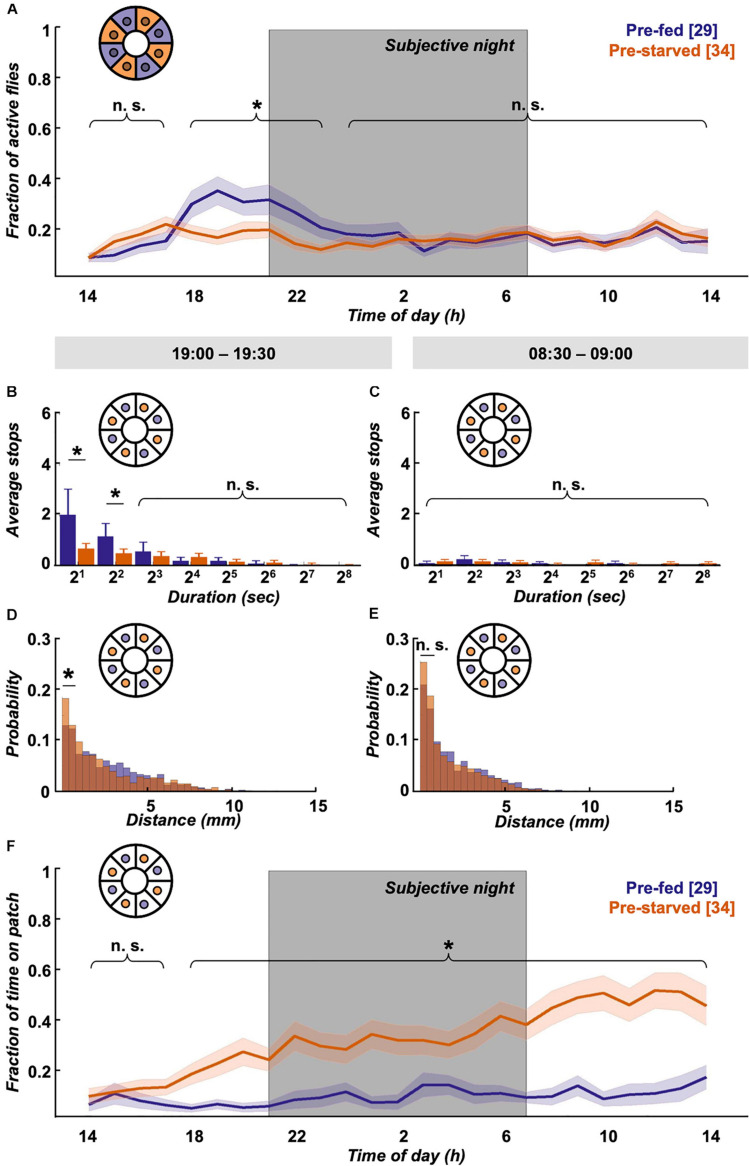
Locomotion activity and food patch preference over 24 h on food patch assay. **(A)** Although initial activity levels on the food patch arena (inset schematic) are comparable, pre-starved flies (orange, *n* = 34) continued to show significantly decreased activity levels during subjective evening and early night, as compared to pre-fed flies (deep purple, *n* = 29). Subjective night indicated by grey-shaded area. There was no significant difference in activity across the rest of the 24 h. **(B)** Pre-fed flies showed significantly increased numbers of stops of shorter duration (2–7 s) on the food patch (inset schematic) than pre-starved flies during peak activity time window of 19:00–19:30, **(C)** whereas no significant difference in food patch stops was observed between 08:30 and 09:00 the next morning between the two groups. *X*-axis tick labels indicate duration of stops with a log 2 scale, where 2^1^ = 2–3 s, 2^2^ = 4–7 s, and so on. **(D)** Short-distance moves (≤2 mm) between both groups were significantly different on the food patch (inset schematic) during the 19:00–19:30 time window, **(E)** but not during the 08:30–09:00 time window. **(F)** Pre-starved flies spent significantly higher fractions of time on the food patch (inset schematic) as compared to pre-fed flies, with exception of the initial 3 h. *Indicate significance levels following Mann–Whitney U-tests (alpha = 0.05), n.s. = not significant. Horizontal lines indicate significance levels between pre-fed and pre-starved flies from a single group. Curly braces represent identical significance levels between pre-fed and pre-starved from multiple groups. *N* values are given in brackets.

Interestingly, despite the quick compensation in feeding motivation, we observe behavioural differences in locomotion between experimental groups in the long term. Pre-fed flies exhibit a characteristic evening activity peak before the subjective night, which is missing in pre-starved flies (Figure 4A). This elevated evening activity in pre-fed flies is accompanied by a significantly higher number of short-stop events in a representative 30 min time window (Figure 4B). On the morning of the next subjective day, stop rates decreased to comparably low levels in both experimental groups (Figure 4C). To see if this bias during the subjective evening is reflected in speed characteristics, we looked at displacement between frames as a proxy for velocity. Indeed, displacement up to 2 mm/s on the food patch in the same time window (19:00–19:30) is significantly different between pre-fed flies and pre-starved flies (Figure 4D). On the subjective next morning (08:30–09:00), movement has slowed down equally in both groups (Figure 4E). Whereas locomotive differences between initially starved and fed flies disappear on the subjective morning next day, another effect in positional preference becomes more pronounced; initially starved flies increasingly prefer to sit on or close to the food patch (Figure 4F).

To summarise, we see that satiation state impacts locomotion and place preference across the day. Within the first hour, pre-starved flies are more active than pre-fed flies, and supposedly compensate their caloric deficit in bouts of short stops until they reach food intake homeostasis. Afterwards, pre-starved flies show reduced activity, particularly during the subjective evening as compared to their pre-fed control group. Interestingly—and despite equivalent hunger motivation—initially starved flies develop increased preference for the food patch over 24 h.

### Initial Starvation State Impacts Movement Speed Over 24 h

**FIGURE 5:**
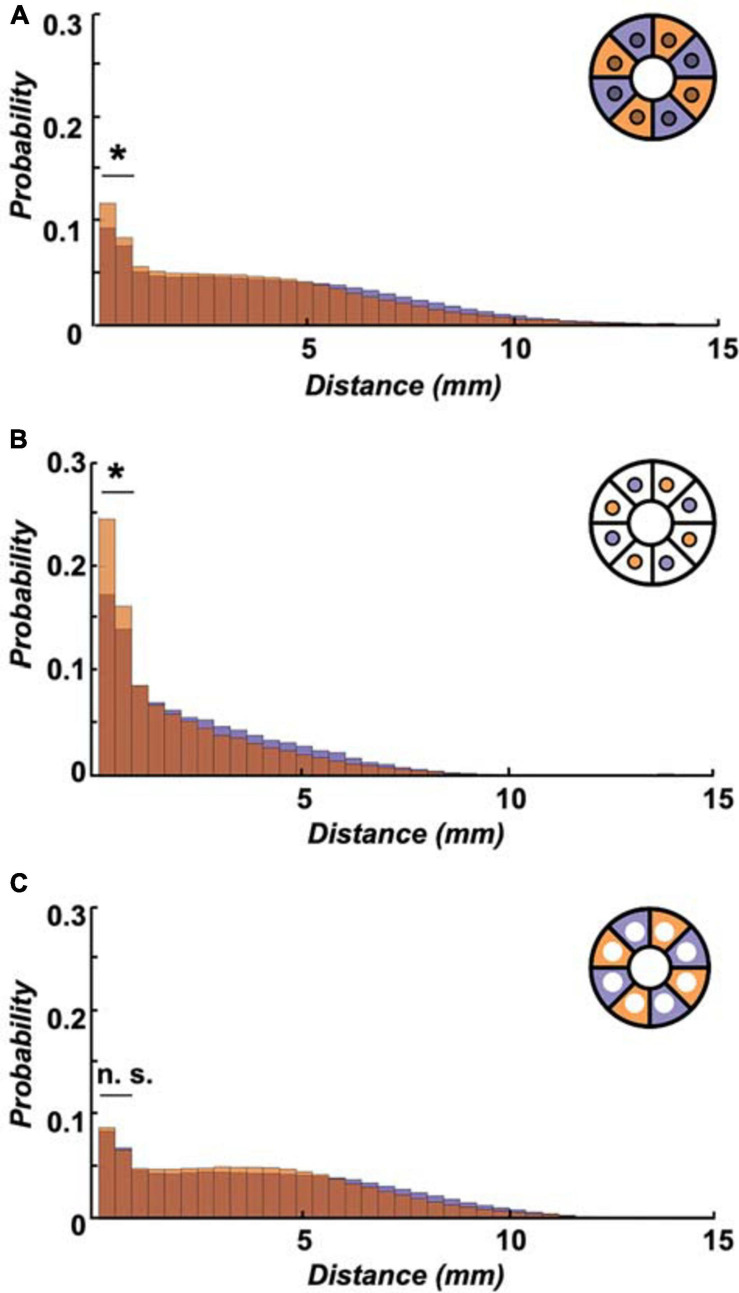
Initially starved flies show more frequent short walks over 24 h. **(A)** Higher probability of short-distance walks (≤2 mm) was observed in pre-starved flies (orange) as compared to pre-fed flies (deep purple) within the entire arena (including food patch area, inset schematic) across 24 h. **(B)** The significantly increased short-distance moves for pre-starved flies than those of pre-fed flies were even more pronounced if analysed on the food patch only (inset schematic). **(C)** Probability of flies showing 0.1–2 mm movements were not significantly different between the two groups as observed in the area outside of the food patch (inset schematic). *Indicate significance levels following Mann–Whitney U-tests (alpha = 0.05), n.s. = not significant. Horizontal lines indicate significance levels between pre-fed and pre-starved flies from a single group.

Initially starved flies are equally active as pre-fed flies for the majority of the observed 24 h timespan (Figure 4A), yet they increasingly confine themselves to the spatially restricted food patch (Figure 4F). How does this impact the flies’ velocity? As an approximation, we examined the displacement distribution across 24 h between both experimental groups, and found that short displacements of up to 2 mm in the arena were significantly more common in pre-starved flies than in pre-fed flies (Figure 5A). These short movements are mostly found to be associated with the food patch (movements on patch itself, as well as movements onto patch or off the patch) (Figure 5B), whereas short movements outside the patch occur with equally low frequency in both experimental groups (Figure 5C). This suggests that both pre-starved and pre-fed flies have comparable internal drives to move, and the location bias of pre-starved flies towards the food patch is compensated by a significantly higher number of short distance moves of up to 2 mm.

### Non-foraging Conditions Reinstate Evening Activity Dynamics in Initially Starved Animals

**FIGURE 6:**
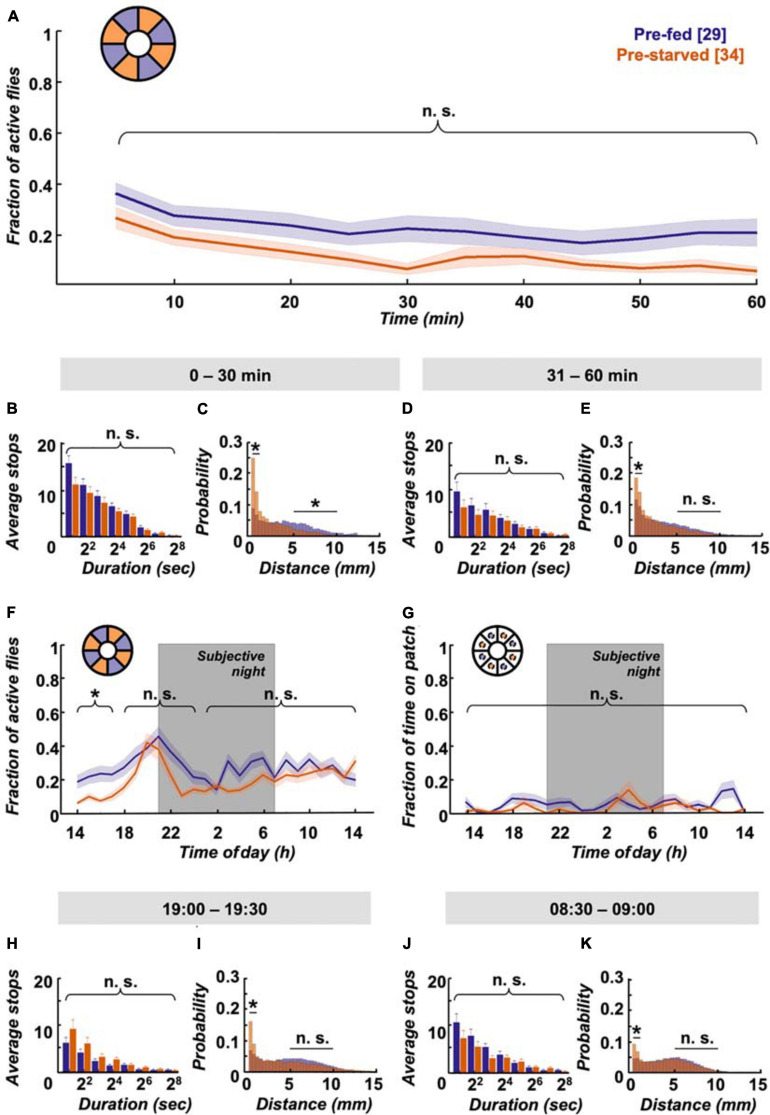
Omnipresent food provision equalises activity levels but not speed. **(A)** Pre-starved (orange, *n* = 27) and pre-fed flies (deep purple, *n* = 34) show no significant difference in activity across the first hour on a homogenous sucrose-covered arena (inset schematic), **(B)** together with no significant differences observed for shorter stops (2–7 s). *X*-axis tick labels indicate duration of stops with a log2 scale, where 2^1^ = 2–3 s, 2^2^ = 4–7 s, and so on. **(C)** However, pre-starved flies did show higher frequency of short feeding-related walks (0–2 mm) in the first 30 min than those of pre-fed flies, while pre-fed flies showed a significantly higher frequency of longer distance walks (5–10 mm). **(D)** There was no significant difference observed again in the next 30 min for shorter stops (2–7 s) between the two fly groups, but **(E)** pre-starved flies continued to show significantly increased short-distance moves as compared to pre-fed flies. **(F)** Pre-starved flies show comparable activity levels to pre-fed flies, including during the evening activity peak, with no significant difference observed across the remaining 24 h. **(G)** Location probability within virtual food patch-sized areas revealed no significant difference in place preference across 24 h for both pre-starved and pre-fed flies. **(H)** During the 19:00–19:30 time window, both groups showed equal number of short stops (2–7 s), with **(I)** consistent higher frequency of short-distance walks seen in pre-starved flies than pre-fed flies. **(J)** No significant difference in short stops (2–7s) was observed during the 08:30–09:00 time window the next morning across both groups, **(K)** and again a significant difference in short-distance moves. Data indicates higher walking speed in pre-fed flies as compared to pre-starved flies. *Indicate significance levels following Mann–Whitney U-tests (alpha = 0.05), n.s. = not significant. Horizontal lines indicate significance levels between pre-fed and pre-starved flies from a single group. Curly braces represent identical significance levels between pre-fed and pre-starved from multiple groups. N values are given in brackets.

A hungry fly has an intrinsic drive to forage and reach satiety. But how does such a fly behave when we take away the need for foraging altogether, when hunger can be satiated anytime, anywhere? In such a context, we adjusted the assay by removing the food patches and instead lacing all inner surfaces with a homogenous layer of 1% agarose containing 200 mM sucrose. The raised initial activity in the food patch assay (Figure 3A) was reduced by about 15% on omnipresent food in pre-fed flies (Figure 6A); with about 50% this effect was even more pronounced in pre-starved flies. The stop lengths in the food-covered arena during the first 30 min are comparable between both experimental groups (Figure 6B), but there is a robust dichotomy in average speed distribution: pre-starved flies preferably move at average speeds up to 2 mm/s, wherein pre-fed flies travel substantial and significant distances at average speeds between 5 and 10 mm/s during this time window (Figure 6C).

During the subsequent 30 min, when initially starved flies supposedly adjusted their caloric needs, stop lengths remain similar (Figure 6D). But while pre-starved flies still significantly prefer slow movements, the preference becomes less pronounced and the shift towards higher average velocities reaches comparable levels between 5 and 10 mm/s in both groups (Figure 6E). The initially lower activity of pre-starved flies as compared to that of pre-fed flies disappears during the remainder of the 24 h experiment duration. In fact, this even includes a reinstated evening activity peak in pre-starved flies (Figure 6F). A virtual food patch (i.e., an area of equivalent size to the food patch) in the omnipresent food arena shows no place preferences for both groups (Figure 6G). It has been reported that flies kept on sugar increase their locomotion in contrast to flies kept on agarose (Lim et al., 2014). Indeed it appears as if overall levels of activity in the omnipresent sucrose arena are slightly elevated, in comparison with fly activity levels in the food patch arena (Figures 4A, 6F). Taken together, ubiquitous food presentation dampens initial hyperactivity during the first 30 min, and restores the activity peak during subjective evening in pre-starved flies.

### Pre-starved Flies Maintain Reduced Walking Speed Across 24 h on Omnipresent Food

As mentioned before, homogenous food distribution in the Panopticon results in equivalent stop dynamics for both pre-starved and pre-fed flies. Indeed, this holds true across all examined time windows (Figures 6B,D,H,J), and is different to what we observed on food patches before (Figures 3B,C, 4B,C). Similar to before however, initially starved flies retain a significant prevalence for slower average speeds up to 2 mm/s (Figures 6C,E,I,K), just as seen in the food patch arena (Figure 5). This reduced average velocity persists even during later time points when both experimental groups likely have comparable satiety levels—and no carbohydrate restrictions whatsoever. Due to its consistency across assays, the reduced average speed seems to be a conserved feature of the flies’ nutritional history. Taken together, this implies a long-lasting effect of starvation experience on average velocity independent of localised food patches.

## Discussion

The Panopticon represents a cost-effective, Petri dish-based locomotion assay which we utilise in two configurations: (i) a classical foraging assay with a single food patch and (ii) an omnipresent food configuration, where every surface in the arena is uniformly covered by substrate. In such a scenario, non-foraging explorative walking behaviour is dissociated from foraging-related search and exploration walking; the flies’ tarsal chemosensors are permanently stimulated and every arena location is equally suited to provide food. Due to constant food provision and humidity buffering, longer-lasting recordings (up and beyond 24 h) are possible. This special layout of our locomotion assay will allow for the further dissemination of activity motivations independent of food localisation, i.e., the drive to find a mate (see below), or initial startle response. This previously described increase in activity leads to increased wall walking, is associated with exploring a new environment and usually lasts for only a very short period (Götz and Biesinger, 1985; Liu et al., 2007). By comparing initial activity in the food patch arena (Figure 3A) and omnipresent food conditions (Figure 6A), it turns out that the startle response is indeed minute in comparison to the foraging activity—or that even fed flies abandon enhanced arena exploration when encountering 200 mM sucrose.

One current caveat of our experiments is that the flies are exclusively resupplied with a carbohydrate food source. Although the flies’ intake target is heavily skewed towards carbohydrates (Lee et al., 2008; Tatar et al., 2014) and flies can survive on sucrose alone for weeks (Hassett, 1948), we cannot rule out additional effects by ongoing protein deprivation or possible micronutrient shortage as shown in other insects (Grund-Mueller et al., 2020). Nonetheless, we only tested male flies which exhibit a more pronounced skew towards sugars than females (Ribeiro and Dickson, 2010). Also, female mating status, egg production and oviposition can affect consumption, as well as food and place choice (Joseph et al., 2009; Carvalho-Santos et al., 2020; Hadjieconomou et al., 2020). Conversely, the drive to find a mate might be the main motivation for the evening activity seen in both pre-fed and pre-starved males in the omnipresent food assay (Figure 6F), although courtship is thought to be associated with activity at dawn (De et al., 2013). Usually, mating drive is assessed as an incentive to engage in courtship (Rings and Goodwin, 2019), but to our knowledge the motivation to locate a female hasn’t been studied in flies (Lee and Wu, 2020); our assay would allow isolating such a locomotion incentive by interfering with known mating motivation circuits (Zhang et al., 2019). If mating drive is indeed the main motivation behind the evening activity, it is superseded by the pre-starved flies’ preference to stay close to a food source (Figure 4A).

Surprisingly, we do not see a pronounced morning activity peak in our assay, as would be expected from circadian DAM assay studies (i.e., De et al., 2013). We cannot entirely explain this, although DAM assays provide higher sensitivity to crepuscular activity (Garbe et al., 2015), and the morning peak can be a subtle component of endogenous rhythmicity in DD lab conditions (Silva et al., 2021). A DD paradigm ensures homogenous illumination during tracking and is less disruptive for internal rhythms than constant light (Green, 1964a). But permanent dark lacks a distinct visual and thermal Zeitgeber signal for morning onset, which, at least under seminatural conditions, has been shown to be the major influence on the morning activity peak, with little contribution from internal clock genes (Green et al., 2015). Furthermore, individuals within a fly population can exhibit crepuscular, diurnal and nocturnal activity pattern (Pegoraro et al., 2020); the tested flies might be skewed in their allele distribution for such circadian traits. The activity peak during the subjective evening however is very robust. Remarkably, this activity peak is only missing in one condition: in pre-starved flies on the food patch Panopticon. Pre-starved flies do ingest increased amounts of food immediately after food is resupplied (Figure 3D). Under constant *ad libitum* food conditions, flies tend to not eat to their maximal capacity but rather maintain an almost empty crop (Edgecomb et al., 1994). A full crop cannot only terminate feeding (Gelperin, 1971; Min et al., 2020; Wang et al., 2020a), but also limit post-prandial explorations (Murata et al., 2017). However, it is doubtful that this “rest-and-digest” effect would last very long after the initial voracious re-feeding period. While pre-fed flies always exhibit the evening activity peak, pre-starved flies—which would be expected to have a ‘rest-and-digest’ period after re-feeding—have a reconstituted evening activity peak under omnipresent food conditions (Figure 6F). This rather indicates a crucial interplay between spatial availability of nutrients and general locomotion motivation across the day to explain the activity differences during the evening in pre-starved flies.

Along with the food patch location preference (Figure 4F), the following picture emerges: it appears that the motivation for pre-starved flies to sit on the food patch could be not to stray too far from a feeding resource, which outcompetes the motivation for raised activity during the subjective evening. As soon as food is omnipresent, the location preference is gone, and evening activity is reinstated. If the place preference in previously starved flies is also triggered by gustatory activation like local food search (Murata et al., 2017) remains to be shown, for example by providing sweet-only food patches (i.e., arabinose) to rule out caloric involvement. It also remains to be shown if ongoing protein deprivation is involved; however, male flies only seek a protein source after days of prolonged protein starvation (Ribeiro and Dickson, 2010).

The second outcome is less obvious, but robust: independent of food patch presence or omnipresent food, the pre-fed flies move faster than pre-starved flies during the course of the experiments (Figures 5, 6C,E,I,K). This could be a delayed effect of the experienced starvation stress; prolonged vibrational stress can lead to reduced voluntary locomotion (Ries et al., 2017). Another possibility could be a long-lasting or even persistent effect on sensory perception (May et al., 2019; Vaziri et al., 2020).

Hunger is known to sensitise certain chemosensory and other circuits at the expense of others, but such sensitisation is usually reversed as soon as the caloric demand is met (Root et al., 2011; Nishimura et al., 2012; Farhan et al., 2013; Inagaki et al., 2014; Longden et al., 2014; Sachse and Beshel, 2016; Grunwald Kadow, 2019; Lin et al., 2019; Wang and Wang, 2019). Habituation and sensitisation of tarsal sugar responses are only described in the minute range (Duerr and Quinn, 1982; Scheiner, 2004; Paranjpe et al., 2012). We assume that such short-lasting effects in each flies’ recurring hunger/feeding/satiety cycles are cancelled out by interindividual variance, since we do not observe any obvious correlation in activity on the binned level of start-synchronised 24 h data (not shown) that would point towards a coordinated habituation effect. Adaptation of taste-sensing neurons can occur over longer time frames, after permanent dietary intervention (May et al., 2019; Wang et al., 2020b). Exposure to high concentration sucrose might either differentially desensitise gustatory sensilla in pre-fed and pre-starved flies, or the prolonged hunger experience in pre-starved flies leads to a permanent sensitisation of gustatory sensilla. In either case, the threshold to sample the substrate would differ between the two experimental groups, resulting in the observed speed differences in the Panopticon.

The most striking phenotype however is the increasing bias of pre-starved flies towards the food patch (Figure 4F). A similar difference in food patch interaction (although not on that timescale) has been observed for sitter and Rover alleles of the *foraging* gene; while Rover flies show normal local search behaviour after ingestion, sitter flies tend to stay close to the food source (Pereira and Sokolowski, 1993). Flies are aware of the food patch position within the arena. This is different from learning the spatial arrangement pattern of food patches, which houseflies seem to be not capable of (Fromm and Bell, 1987). But flies can remember locations and learn to efficiently navigate towards previously encountered targets like visual landmarks (Neuser et al., 2008), safe spots (Ofstad et al., 2011), or food sources (Navawongse et al., 2016), even in the dark and without usage of visual or olfactory sense (Kim and Dickinson, 2017).

It appears that the biogenic amine serotonin (5-HT) is involved in such place learning (Sitaraman et al., 2008, 2017; Sitaraman and LaFerriere, 2020). Furthermore, different 5-HT subsets or 5-HT regulation interfere with feeding (French et al., 2014; Albin et al., 2015; Liu et al., 2015), food seeking behaviour (He et al., 2020), locomotion (Yellman et al., 1997; Howard et al., 2019), sleep architecture (Liu et al., 2019), and quiescence (Pooryasin and Fiala, 2015). Thus, 5-HT manipulation provides a good candidate for further studies in the Panopticon (Tierney, 2020).

Similarly, octopamine (OA) and tyramine (TA) influence locomotion in a state-dependent manner; starvation shifts the OA/TA balance via TBH expression levels and leads to hunger-induced hyperactivity (Yang et al., 2015; Schützler et al., 2019). A single OA neuron signals satiation and stops food-motivated search (Sayin et al., 2019), and the same neuron can initiate feeding behaviour (Youn et al., 2018). Also, OA influences AKH signalling for diurnal pattern generation (Pauls et al., 2020), and might be affected in pre-starved flies during the blocked evening activity peak. Given the pleiotropic actions of OA, place preference may be impacted as well (Selcho and Pauls, 2019).

The food patch location is most likely associated with food reward in both pre-fed and pre-starved flies, analogous to odour associations with caloric value (Burke and Waddell, 2011; Fujita and Tanimura, 2011; Huetteroth et al., 2015; Ichinose et al., 2015; Musso et al., 2015; Yamagata et al., 2015; Zhang et al., 2015; Das et al., 2016). Such long-lasting, food-related odour memories are stored in the mushroom body (MB) (Krashes and Waddell, 2008), and it is clear that this structure, especially its zonal dopaminergic modulatory innervation, has a central instructional role in motivational foraging and feeding (Tsao et al., 2018; Musso et al., 2019; May et al., 2020).

In the Panopticon experiment, the food-place association could be enforced by two factors in pre-starved flies: First, the absolute amount of food that is ingested by pre-starved flies within the first 30 min is bigger, since they supposedly need to cover their caloric deficit (Figure 3D). Secondly, the lack of caloric signals during starvation renders food-associative MB circuits particularly sensitive to the next food encounter (Hirano and Saitoe, 2013; Hirano et al., 2013, 2016; Plaçais et al., 2017; Wu et al., 2018). For example, starved flies, contrary to fed flies, do not require additional sleep to consolidate a food-odour association (Chouhan et al., 2020). It appears the subjectively perceived value of food is higher in starved flies than in fed flies. So during this time of transition in a new environment, both quantity and perceived quality of the ingested food on the food patch would be higher for pre-starved flies. These two effects together could lead to a strong and long-lasting positive association with the food patch location that influences location decision making beyond nutritional demand for the subsequent 24 h.

However, some issues are not addressed by this explanation. Retrieval of a food-associated memory depends on the motivational incentive of hunger; a starved fly will utilise an olfactory food association to increase its chances to feed, whereas fed flies will only do so after being starved once more (Krashes and Waddell, 2008). Similarly, starved flies would have a higher incentive to retrieve and use their place memory of the food patch location, and indeed, starved flies exhibit a higher and more frequent return rate to a known food patch, be it real or virtual (Corrales-Carvajal et al., 2016; Kim and Dickinson, 2017; Murata et al., 2017; Corfas et al., 2019; Haberkern et al., 2019).

In this regard it is unlikely that the lasting food patch preference in the Panopticon depends on concurrent hunger as the motivational drive to retrieve spatial memory. All other food-related behaviours like activity, stop distribution or sip number are aligned between pre-starved and pre-fed flies within an hour, indicating comparable hunger motivation from then onwards (Figure 3), and protein hunger only starts to influence male food choice much later (Ribeiro and Dickson, 2010). It also needs to be taken into account that food association in the Panopticon is not formed with odours but with place, and associations of spatial features involve the central complex (Liu et al., 2006; Stern et al., 2019); it is equally possible that plastic changes in this structure contribute to the observed location preference. In a place learning assay, the unavoidability of an aversive heat stimulus can boost its reinforcing propensities (Sitaraman and Zars, 2010); it is feasible that the same is the case for unavoidable starvation.

But why does this effect appear to become stronger over time? It might be possible that the pre-starved flies generalise from the starvation experience, and hence seek proximity to the food source. Increasing generalisation of an aversive stimulus over time is not only observed for odour-shock learning and male aggression in flies (König et al., 2017; Kim et al., 2018), but is also a characteristic feature of post-traumatic stress disorders in both animals and humans (Stam, 2007). A similar long-lasting effect has been described for predator-induced oviposition preference (Kacsoh et al., 2015). Here, gravid female flies are exposed to parasitoid wasps for several hours. After removing the wasps, the females choose ethanol-laced patches over control patches for days (fly larvae have a higher ethanol tolerance than wasp larvae). As in the Panopticon, prolonged exposure to a distressing stimulus (hunger or parasitoid wasps) influences a later choice (place preference) even after the stressor was removed. Interestingly, MB inhibition and several memory mutants abolished this long-lasting skew exclusively after wasp removal, but not under immediate threat; it will be interesting to see how MB function and memory genes impact the place preference on the Panopticon.

## Outlook

We present here a new paradigm to examine locomotion behaviour and place preference, under foraging conditions (food patch Panopticon) or under permanent chemosensory stimulation (omnipresent food Panopticon). The food patch Panopticon will help to examine the neuronal circuits underlying long-lasting effects of starvation on place preference, and how this apparently non-associative process relates to known associative long-lasting memory function.

In the omnipresent food Panopticon, we will be able to assess the influence of state-modulating or state-mediating substances like biogenic amines or neuropeptides and their receptors on locomotion parameters, without interference of foraging-motivated movement. Being able to do this over prolonged periods will help to discern long-lasting pleiotropic effects of these effectors (Martelli et al., 2017; Dreyer et al., 2019; Nässel et al., 2019; Pauls et al., 2020).

## Data Availability Statement

The raw data supporting the conclusions of this article will be made available by the authors, without undue reservation.

## Author Contributions

DM, TT, and WH conceived and designed the experiments, analysed the results, and wrote the manuscript. DM and RH performed the experiments. DM and TT wrote the MATLAB script. All authors provided comments and approved the manuscript.

## Conflict of Interest

The authors declare that the research was conducted in the absence of any commercial or financial relationships that could be construed as a potential conflict of interest.

## Footnotes

1 https://github.com/dmahishi/Panopticon.git

## Supplementary Material

Supplementary Figure 1 Representative plots for multi-step quality check. **(A)** Temporal distribution of failed/missed detections with total error rate (%) indicated on the right panel for individual flies/sectors across 24 h. **(B)** Walking path traces for the entire arena divided by sectors, with **(C)** 3D representation, expandable on the *Z*-axis representing walking traces across the total duration of activity recording for 24 h. Time scales represented as frame numbers (@ 1 frame/s). Blue-yellow-red colour transition represents walking traces from 0-n frame count (across total duration of the experiment). **(D)** To visually identify long-distance movements associated with potentially false-positive detections, corresponding images for top ten frames with flies covering the longest distances can be extracted (example image for Sector 8). Such images include up to 2 consecutive long distance moves as indicated by individually coloured walking trace lines and the fly itself encircled with the respective colour. Apart from the queried sector, all the other sectors also indicate distances moves in corresponding frames.

## References

[B1] Albin S. D. Kaun K. R. Knapp J.-M. Chung P. Heberlein U. Simpson J. H. (2015). A Subset of Serotonergic Neurons Evokes Hunger in Adult Drosophila. *Curr. Biol.* 25 2435–2440. 10.1016/j.cub.2015.08.005 26344091

[B2] Barwell T. Raina S. Seroude L. (2020). Versatile method to measure locomotion in adult *Drosophila*. *Genome* 2020 1–7. 10.1139/gen-2020-0044 32552119

[B3] Behbahani A. H. Palmer E. H. Corfas R. A. Dickinson M. H. (2021). *Drosophila* local search emerges from iterative odometry of consecutive run lengths. *bioRxiv* 10.1101/2021.01.18.427191

[B4] Bell W. J. (1990). Searching Behavior Patterns in Insects. *Annu. Rev. Entomol.* 35 447–467. 10.1146/annurev.en.35.010190.002311

[B5] Bell W. J. Cathy T. Roggero R. J. Kipp L. R. Tobin T. R. (1985). Sucrose-stimulated searching behaviour of Drosophila melanogaster in a uniform habitat: modulation by period of deprivation. *Animal. Behaviour.* 33 436–448. 10.1016/S0003-3472(85)80068-3

[B6] Brockmann A. Basu P. Shakeel M. Murata S. Murashima N. Boyapati R. K. (2018). Sugar Intake Elicits Intelligent Searching Behavior in Flies and Honey Bees. *Front. Behav. Neurosci.* 12:280. 10.3389/fnbeh.2018.00280 30546299 PMC6279864

[B7] Burke C. J. Waddell S. (2011). Remembering Nutrient Quality of Sugar in Drosophila. *Curr. Biol.* 21 746–750. 10.1016/j.cub.2011.03.032 21514159 PMC3094154

[B8] Campbell R. (2020). *raacampbell**/shadedErrorBar.* Available online at: https://github.com/raacampbell/shadedErrorBar: Github (accessed April 30, 2020).

[B9] Carvalho G. B. Kapahi P. Benzer S. (2005). Compensatory ingestion upon dietary restriction in Drosophila melanogaster. *Nat. Methods* 2 813–815. 10.1038/nmeth798 16278649 PMC2745347

[B10] Carvalho-Santos Z. Cardoso-Figueiredo R. Elias A. P. Tastekin I. Baltazar C. Ribeiro C. (2020). Cellular metabolic reprogramming controls sugar appetite in Drosophila. *Nat. Metab.* 2 958–973. 10.1038/s42255-020-0266-x 32868922

[B11] Chouhan N. S. Griffith L. C. Haynes P. Sehgal A. (2020). Availability of food determines the need for sleep in memory consolidation. *Nature* 2020 2997–y. 10.1038/s41586-020-2997-y 33268891 PMC7856038

[B12] Connolly K. (1966a). Locomotor activity in Drosophila. II. Selection for active and inactive strains. *Anim. Behav.* 14 444–449. 10.1016/S0003-3472(66)80043-X 5972802

[B13] Connolly K. J. (1966b). Locomotor Activity in Drosophila as a Function of Food Deprivation. *Nature* 209 224–224. 10.1038/209224a0 5912441

[B14] Corfas R. A. Sharma T. Dickinson M. H. (2019). Diverse Food-Sensing Neurons Trigger Idiothetic Local Search in Drosophila. *Curr. Biol.* 29 1660.e–1668.e. 10.1016/j.cub.2019.03.004 31056390 PMC6532790

[B15] Corrales-Carvajal V. M. Faisal A. A. Ribeiro C. (2016). Internal states drive nutrient homeostasis by modulating exploration-exploitation trade-off. *eLife* 5:e19920. 10.7554/eLife.19920 27770569 PMC5108593

[B16] Das G. Lin S. Waddell S. (2016). Remembering Components of Food in Drosophila. *Front. Integrat. Neurosci.* 10:00004. 10.3389/fnint.2016.00004 26924969 PMC4759284

[B17] Davies L. R. Schou M. F. Kristensen T. N. Loeschcke V. (2018). Linking developmental diet to adult foraging choice in *Drosophila melanogaster*. *J. Exp. Biol.* 221:jeb175554. 10.1242/jeb.175554 29666197

[B18] De J. Varma V. Saha S. Sheeba V. Sharma V. K. (2013). Significance of activity peaks in fruit flies, Drosophila melanogaster, under seminatural conditions. *Proc. Natl. Acad. Sci.* 110 8984–8989. 10.1073/pnas.1220960110 23671102 PMC3670394

[B19] Dethier V. G. (1957). Communication by Insects ^∗^. *Physiol. Dancini.* 125:6. 10.1126/science.125.3243.331 17794437

[B20] Dethier V. G. (1976). *The hungry fly: a physiological study of the behavior associated with feeding.* Cambridge, MA: Harvard University Press.

[B21] Donelson N. Kim E. Z. Slawson J. B. Vecsey C. G. Huber R. Griffith L. C. (2012). High-Resolution Positional Tracking for Long-Term Analysis of Drosophila Sleep and Locomotion Using the “Tracker” Program. *PLoS One* 7:e37250. 10.1371/journal.pone.0037250 22615954 PMC3352887

[B22] Dreyer A. P. Martin M. M. Fulgham C. V. Jabr D. A. Bai L. Beshel J. (2019). A circadian output center controlling feeding:fasting rhythms in Drosophila. *PLoS Genet.* 15:e1008478. 10.1371/journal.pgen.1008478 31693685 PMC6860455

[B23] Duerr J. S. Quinn W. G. (1982). Three Drosophila mutations that block associative learning also affect habituation and sensitization. *Proc. Natl. Acad. Sci. U S A* 79 3646–3650. 10.1073/pnas.79.11.3646 6808513 PMC346480

[B24] Dus M. Min S. Keene A. C. Lee G. Y. Suh G. S. B. (2011). Taste-independent detection of the caloric content of sugar in Drosophila. *Proc. Natl. Acad. Sci. U.S.A.* 108 11644–11649. 10.1073/pnas.1017096108 21709242 PMC3136275

[B25] Edgecomb R. S. Harth C. E. Schneiderman A. M. (1994). Regulation of feeding behavior in adult Drosophila melanogaster varies with feeding regime and nutritional state. *J. Exp. Biol.* 197 215–235. 7852903 10.1242/jeb.197.1.215

[B26] Farhan A. Gulati J. Groβe-Wilde E. Vogel H. Hansson B. S. Knaden M. (2013). The CCHamide 1 receptor modulates sensory perception and olfactory behavior in starved Drosophila. *Sci. Rep.* 3:2765. 10.1038/srep02765 24067446 PMC3783891

[B27] French A. S. Simcock K. L. Rolke D. Gartside S. E. Blenau W. Wright G. A. (2014). The role of serotonin in feeding and gut contractions in the honeybee. *J. Physiol.* 61 8–15. 10.1016/j.jinsphys.2013.12.005 24374107 PMC3969292

[B28] Fromm J. E. Bell W. J. (1987). Search orientation of Musca domestica in patches of sucrose drops. *Physiol. Entomol.* 12 297–307. 10.1111/j.1365-3032.1987.tb00754.x

[B29] Fujita M. Tanimura T. (2011). Drosophila Evaluates and Learns the Nutritional Value of Sugars. *Curr. Biol.* 21 751–755. 10.1016/j.cub.2011.03.058 21514154

[B30] Garbe D. S. Bollinger W. L. Vigderman A. Masek P. Gertowski J. Sehgal A. (2015). Context-specific comparison of sleep acquisition systems in Drosophila. *Biol. Open* 4 1558–1568. 10.1242/bio.013011 26519516 PMC4728345

[B31] Gelperin A. (1971). Abdominal sensory neurons providing negative feedback to the feeding behavior of the blowfly. *Z. Vergl. Physiol.* 72 17–31. 10.1007/BF00299201

[B32] Götz K. G. Biesinger R. (1985). Centrophobism inDrosophila melanogaster: II. Physiological approach to search and search control. *J. Comp. Physiol.* 156 329–337. 10.1007/BF00610726

[B33] Green E. W. O’Callaghan E. K. Hansen C. N. Bastianello S. Bhutani S. Vanin S. (2015). *Drosophila* circadian rhythms in seminatural environments: Summer afternoon component is not an artifact and requires TrpA1 channels. *Proc. Natl. Acad. Sci. USA* 112 8702–8707. 10.1073/pnas.1506093112 26124142 PMC4507215

[B34] Green G. W. (1964a). The control of spontaneous locomotor activity in Phormia regina Meigen—I. Locomotor activity patterns of intact flies. *J. Insect Physiol.* 10 711–726. 10.1016/0022-1910(64)90054-X

[B35] Green G. W. (1964b). The control of spontaneous locomotor activity in Phormia regina Meigen—II. Experiments to determine the mechanism involved. *J. Insect Physiol.* 10 727–752. 10.1016/0022-1910(64)90055-1

[B36] Grund-Mueller N. Ruedenauer F. A. Spaethe J. Leonhardt S. D. (2020). Adding Amino Acids to a Sucrose Diet Is Not Sufficient to Support Longevity of Adult Bumble Bees. *Insects* 11:247. 10.3390/insects11040247 32326445 PMC7240467

[B37] Grunwald Kadow I. C. (2019). State-dependent plasticity of innate behavior in fruit flies. *Curr. Opin. Neurobiol.* 54 60–65. 10.1016/j.conb.2018.08.014 30219668

[B38] Guo F. Yu J. Jung H. J. Abruzzi K. C. Luo W. Griffith L. C. (2016). Circadian neuron feedback controls the Drosophila sleep–activity profile. *Nature* 536 292–297. 10.1038/nature19097 27479324 PMC5247284

[B39] Haberkern H. Basnak M. A. Ahanonu B. Schauder D. Cohen J. D. Bolstad M. (2019). Visually Guided Behavior and Optogenetically Induced Learning in Head-Fixed Flies Exploring a Virtual Landscape. *Curr. Biol.* 29 1647.e–1659.e. 10.1016/j.cub.2019.04.033 31056392

[B40] Hadjieconomou D. King G. Gaspar P. Mineo A. Blackie L. Ameku T. (2020). Enteric neurons increase maternal food intake during reproduction. *Nature* 587 455–459. 10.1038/s41586-020-2866-8 33116314 PMC7610780

[B41] Hassett C. C. (1948). THE UTILIZATION OF SUGARS AND OTHER SUBSTANCES BY DROSOPHILA. *Biol. Bull.* 95 114–123. 10.2307/1538158 18874957

[B42] He J. Hommen F. Lauer N. Balmert S. Scholz H. (2020). Serotonin transporter dependent modulation of food-seeking behavior. *PLoS One* 15:e0227554. 10.1371/journal.pone.0227554 31978073 PMC6980608

[B43] Hirano Y. Ihara K. Masuda T. Yamamoto T. Iwata I. Takahashi A. (2016). Shifting transcriptional machinery is required for long-term memory maintenance and modification in Drosophila mushroom bodies. *Nat. Commun.* 7:13471. 10.1038/ncomms13471 27841260 PMC5114576

[B44] Hirano Y. Masuda T. Naganos S. Matsuno M. Ueno K. Miyashita T. (2013). Fasting launches CRTC to facilitate long-term memory formation in Drosophila. *Science* 339 443–446. 10.1126/science.1227170 23349290

[B45] Hirano Y. Saitoe M. (2013). Hunger and memory; CRTC coordinates long-term memory with the physiological state, hunger. *Commun. Integr. Biol.* 6:e25152. 10.4161/cib.25152 24265850 PMC3829949

[B46] Howard C. E. Chen C.-L. Tabachnik T. Hormigo R. Ramdya P. Mann R. S. (2019). Serotonergic Modulation of Walking in Drosophila. *Curr. Biol.* 29 4218.e–4230.e. 10.1016/j.cub.2019.10.042 31786064 PMC6935052

[B47] Huetteroth W. Perisse E. Lin S. Klappenbach M. Burke C. Waddell S. (2015). Sweet Taste and Nutrient Value Subdivide Rewarding Dopaminergic Neurons in Drosophila. *Curr. Biol.* 25 751–758. 10.1016/j.cub.2015.01.036 25728694 PMC4372253

[B48] Hughson B. N. Anreiter I. Jackson Chornenki N. L. Murphy K. R. Ja W. W. Huber R. (2018). The adult foraging assay (AFA) detects strain and food-deprivation effects in feeding-related traits of Drosophila melanogaster. *J. Insect Physiol.* 106 20–29. 10.1016/j.jinsphys.2017.08.011 28860037 PMC5832525

[B49] Ichinose T. Aso Y. Yamagata N. Abe A. Rubin G. M. Tanimoto H. (2015). Reward signal in a recurrent circuit drives appetitive long-term memory formation. *eLife* 4:e10719. 10.7554/eLife.10719 26573957 PMC4643015

[B50] Inagaki H. K. Panse K. M. Anderson D. J. (2014). Independent, reciprocal neuromodulatory control of sweet and bitter taste sensitivity during starvation in Drosophila. *Neuron* 84 806–820. 10.1016/j.neuron.2014.09.032 25451195 PMC4365050

[B51] Itskov P. M. Moreira J.-M. Vinnik E. Lopes G. Safarik S. Dickinson M. H. (2014). Automated monitoring and quantitative analysis of feeding behaviour in Drosophila. *Nat. Commun.* 5:4560. 10.1038/ncomms5560 25087594 PMC4143931

[B52] Joseph R. M. Devineni A. V. King I. F. G. Heberlein U. (2009). Oviposition preference for and positional avoidance of acetic acid provide a model for competing behavioral drives in Drosophila. *Proc. Natl. Acad. Sci.* 106 11352–11357. 10.1073/pnas.0901419106 19541615 PMC2698888

[B53] Kacsoh B. Z. Bozler J. Hodge S. Ramaswami M. Bosco G. (2015). A Novel Paradigm for Nonassociative Long-Term Memory in *Drosophila*: Predator-Induced Changes in Oviposition Behavior. *Genetics* 199 1143–1157. 10.1534/genetics.114.172221 25633088 PMC4391557

[B54] Kim I. S. Dickinson M. H. (2017). Idiothetic Path Integration in the Fruit Fly Drosophila melanogaster. *Curr. Biol.* 27 2227.e–2238.e. 10.1016/j.cub.2017.06.026 28736164

[B55] Kim Y.-K. Saver M. Simon J. Kent C. F. Shao L. Eddison M. (2018). Repetitive aggressive encounters generate a long-lasting internal state in *Drosophila melanogaster* males. *Proc. Natl. Acad. Sci. USA* 115 1099–1104. 10.1073/pnas.1716612115 29339481 PMC5798363

[B56] Knoppien P. Jan N. C. van der Pers van Delden W. (2000). Quantification of Locomotion and the Effect of Food Deprivation on Locomotor Activity in Drosophila. *J. Insect Behav.* 13 27–43.

[B57] König C. Antwi-Adjei E. Ganesan M. Kilonzo K. Viswanathan V. Durairaja A. (2017). Aversive olfactory associative memory loses odor specificity over time. *J. Exp. Biol.* 220 1548–1553. 10.1242/jeb.155317 28468811 PMC5450803

[B58] Krashes M. J. Waddell S. (2008). Rapid Consolidation to a radish and Protein Synthesis-Dependent Long-Term Memory after Single-Session Appetitive Olfactory Conditioning in Drosophila. *J. Neurosci.* 28 3103–3113. 10.1523/JNEUROSCI.5333-07.2008 18354013 PMC2516741

[B59] Landayan D. Feldman D. S. Wolf F. W. (2018). Satiation state-dependent dopaminergic control of foraging in Drosophila. *Sci. Rep.* 8:5777. 10.1038/s41598-018-24217-1 29636522 PMC5893590

[B60] Lee K. P. Simpson S. J. Clissold F. J. Brooks R. Ballard J. W. O. Taylor P. W. (2008). Lifespan and reproduction in Drosophila: New insights from nutritional geometry. *Proc. Natl. Acad.* 105 2498–2503. 10.1073/pnas.0710787105 18268352 PMC2268165

[B61] Lee S. S. Wu M. N. (2020). Neural circuit mechanisms encoding motivational states in Drosophila. *Curr. Opin. Neurobiol.* 64 135–142. 10.1016/j.conb.2020.05.002 32563845 PMC7669672

[B62] Lim R. S. Eyjólfsdóttir E. Shin E. Perona P. Anderson D. J. (2014). How Food Controls Aggression in Drosophila. *PLoS One* 9:e105626. 10.1371/journal.pone.0105626 25162609 PMC4146546

[B63] Lin S. Senapati B. Tsao C.-H. (2019). Neural basis of hunger-driven behaviour in *Drosophila*. *Open Biol.* 9 180259. 10.1098/rsob.180259 30914005 PMC6451361

[B64] Liu C. Meng Z. Wiggin T. D. Yu J. Reed M. L. Guo F. (2019). A Serotonin-Modulated Circuit Controls Sleep Architecture to Regulate Cognitive Function Independent of Total Sleep in Drosophila. *Curr. Biol.* 29 3635.e–3646.e. 10.1016/j.cub.2019.08.079 31668619 PMC6832866

[B65] Liu G. Seiler H. Wen A. Zars T. Ito K. Wolf R. (2006). Distinct memory traces for two visual features in the Drosophila brain. *Nature* 439 551–556. 10.1038/nature04381 16452971

[B66] Liu L. Davis R. L. Roman G. (2007). Exploratory Activity in Drosophila Requires the *kurtz* Nonvisual Arrestin. *Genetics* 175 1197–1212. 10.1534/genetics.106.068411 17151232 PMC1840054

[B67] Liu Y. Luo J. Carlsson M. A. Nässel D. R. (2015). Serotonin and insulin-like peptides modulate leucokinin-producing neurons that affect feeding and water homeostasis in *Drosophila*: Modulation of LK Neurons in *Drosophila*. *J. Comparat. Neurol.* 523 1840–1863. 10.1002/cne.23768 25732325

[B68] Longden K. D. Muzzu T. Cook D. J. Schultz S. R. Krapp H. G. (2014). Nutritional State Modulates the Neural Processing of Visual Motion. *Curr. Biol.* 24 890–895. 10.1016/j.cub.2014.03.005 24684935

[B69] Mahishi D. Huetteroth W. (2019). The prandial process in flies. *Curr. Opin. Insect Sci.* 36 157–166. 10.1016/j.cois.2019.09.004 31765996

[B70] Mann K. Gordon M. D. Scott K. (2013). A Pair of Interneurons Influences the Choice between Feeding and Locomotion in Drosophila. *Neuron* 79 754–765. 10.1016/j.neuron.2013.06.018 23972600 PMC3750742

[B71] Martelli C. Pech U. Kobbenbring S. Pauls D. Bahl B. Sommer M. V. (2017). SIFamide Translates Hunger Signals into Appetitive and Feeding Behavior in Drosophila. *Cell Reports* 20 464–478. 28700946 10.1016/j.celrep.2017.06.043

[B72] Martin J.-R. (2004). A portrait of locomotor behaviour in Drosophila determined by a video-tracking paradigm. *Behav. Proces.* 67 207–219. 10.1016/j.beproc.2004.04.003 15240058

[B73] May C. E. Rosander J. Gottfried J. Dennis E. Dus M. (2020). Dietary sugar inhibits satiation by decreasing the central processing of sweet taste. *eLife* 9:e54530. 10.7554/eLife.54530 32539934 PMC7297538

[B74] May C. E. Vaziri A. Lin Y. Q. Grushko O. Khabiri M. Wang Q.-P. (2019). High Dietary Sugar Reshapes Sweet Taste to Promote Feeding Behavior in Drosophila melanogaster. *Cell Reports* 27 1675.e–1685.e. 10.1016/j.celrep.2019.04.027 31067455 PMC6561488

[B75] Meunier N. Belgacem Y. H. Martin J.-R. (2007). Regulation of feeding behaviour and locomotor activity by takeout in Drosophila. *J. Exp. Biol.* 210 1424–1434. 10.1242/jeb.02755 17401125

[B76] Min S. Oh Y. Verma P. Van Vactor D. Suh G. S. B. Liberles S. D. (2020). Control of feeding by Piezo-mediated gut mechanosensation in *Drosophila*. *Neuroscience* 2020 293712. 10.1101/2020.09.11.293712 PMC7920550 33599608

[B77] Moreira J.-M. Itskov P. M. Goldschmidt D. Baltazar C. Steck K. Tastekin I. (2019). optoPAD, a closed-loop optogenetics system to study the circuit basis of feeding behaviors. *eLife* 8:e43924. 10.7554/eLife.43924 31226244 PMC6589098

[B78] Murata S. Brockmann A. Tanimura T. (2017). Pharyngeal stimulation with sugar triggers local searching behavior in *Drosophila*. *J. Exp. Biol.* 220 3231–3237. 10.1242/jeb.161646 28684466

[B79] Murphy K. R. Deshpande S. A. Yurgel M. E. Quinn J. P. Weissbach J. L. Keene A. C. (2016). Postprandial sleep mechanics in Drosophila. *eLife* 5:e19334. 10.7554/eLife.19334 27873574 PMC5119887

[B80] Musso P.-Y. Junca P. Jelen M. Feldman-Kiss D. Zhang H. Chan R. C. (2019). Closed-loop optogenetic activation of peripheral or central neurons modulates feeding in freely moving Drosophila. *eLife* 8:e45636. 10.7554/eLife.45636 31322499 PMC6668987

[B81] Musso P.-Y. Lampin-Saint-Amaux A. Tchenio P. Preat T. (2017). Ingestion of artificial sweeteners leads to caloric frustration memory in Drosophila. *Nat. Commun.* 8:1803. 10.1038/s41467-017-01989-0 29180783 PMC5703724

[B82] Musso P.-Y. Tchenio P. Preat T. (2015). Delayed Dopamine Signaling of Energy Level Builds Appetitive Long-Term Memory in Drosophila. *Cell Reports* 10 1023–1031. 10.1016/j.celrep.2015.01.036 25704807

[B83] Nässel D. R. Pauls D. Huetteroth W. (2019). Neuropeptides in modulation of Drosophila behavior: how to get a grip on their pleiotropic actions. *Curr. Opin. Insect Sci.* 36 1–8. 10.1016/j.cois.2019.03.002 31280184

[B84] Navawongse R. Choudhury D. Raczkowska M. Stewart J. C. Lim T. Rahman M. (2016). Drosophila learn efficient paths to a food source. *Neurobiol. Learn. Memory* 131 176–181. 10.1016/j.nlm.2016.03.019 27063671

[B85] Neuser K. Triphan T. Mronz M. Poeck B. Strauss R. (2008). Analysis of a spatial orientation memory in Drosophila. *Nature* 453 1244–1247. 10.1038/nature07003 18509336

[B86] Nishimura A. Ishida Y. Takahashi A. Okamoto H. Sakabe M. Itoh M. (2012). Starvation-Induced Elevation of Taste Responsiveness and Expression of a Sugar Taste Receptor Gene in *Drosophila melanogaster*. *J. Neurogenet.* 26 206–215. 10.3109/01677063.2012.694931 22794108

[B87] Ofstad T. A. Zuker C. S. Reiser M. B. (2011). Visual place learning in Drosophila melanogaster. *Nature* 474 204–207. 10.1038/nature10131 21654803 PMC3169673

[B88] Paranjpe P. Rodrigues V. VijayRaghavan K. Ramaswami M. (2012). Gustatory habituation in Drosophila relies on rutabaga (adenylate cyclase)-dependent plasticity of GABAergic inhibitory neurons. *Learn. Memory* 19 627–635. 10.1101/lm.026641.112 23169996

[B89] Park J. H. Carvalho G. B. Murphy K. R. Ehrlich M. R. Ja W. W. (2017). Sucralose Suppresses Food Intake. *Cell Metab.* 25 484–485. 10.1016/j.cmet.2017.02.011 28273467 PMC5384720

[B90] Pauls D. Selcho M. Räderscheidt J. Amatobi K. Fekete A. Krischke M. (2020). Endocrine fine-tuning of daily locomotor activity patterns under non-starving conditions in Drosophila. *bioRxiv* 2020:947556. 10.1101/2020.02.13.947556

[B91] Pegoraro M. Flavell L. M. M. Menegazzi P. Colombi P. Dao P. Helfrich-Förster C. (2020). The genetic basis of diurnal preference in Drosophila melanogaster. *BMC Genomics* 21:596. 10.1186/s12864-020-07020-z 32862827 PMC7457780

[B92] Pereira H. S. Sokolowski M. B. (1993). Mutations in the larval foraging gene affect adult locomotory behavior after feeding in Drosophila melanogaster. *Proc. Natl. Acad. Sci.* 90 5044–5046. 10.1073/pnas.90.11.5044 8506349 PMC46650

[B93] Plaçais P.-Y. de Tredern É Scheunemann L. Trannoy S. Goguel V. Han K.-A. (2017). Upregulated energy metabolism in the Drosophila mushroom body is the trigger for long-term memory. *Nat. Commun.* 8:15510. 10.1038/ncomms15510 28580949 PMC5465319

[B94] Pooryasin A. Fiala A. (2015). Identified Serotonin-Releasing Neurons Induce Behavioral Quiescence and Suppress Mating in Drosophila. *J. Neurosci.* 35 12792–12812. 10.1523/JNEUROSCI.1638-15.2015 26377467 PMC6795202

[B95] Ribeiro C. Dickson B. J. (2010). Sex Peptide Receptor and Neuronal TOR/S6K Signaling Modulate Nutrient Balancing in Drosophila. *Curr. Biol.* 20 1000–1005. 10.1016/j.cub.2010.03.061 20471268

[B96] Ries A.-S. Hermanns T. Poeck B. Strauss R. (2017). Serotonin modulates a depression-like state in Drosophila responsive to lithium treatment. *Nat. Commun.* 8:15738. 10.1038/ncomms15738 28585544 PMC5467214

[B97] Rings A. Goodwin S. F. (2019). To court or not to court – a multimodal sensory decision in Drosophila males. *Curr. Opin. Insect Sci.* 35 48–53. 10.1016/j.cois.2019.06.009 31336357

[B98] Robie A. A. Straw A. D. Dickinson M. H. (2010). Object preference by walking fruit flies, Drosophila melanogaster, is mediated by vision and graviperception. *J. Exp. Biol.* 213 2494–2506. 10.1242/jeb.041749 20581279 PMC2892423

[B99] Root C. M. Ko K. I. Jafari A. Wang J. W. (2011). Presynaptic Facilitation by Neuropeptide Signaling Mediates Odor-Driven Food Search. *Cell* 145 133–144. 10.1016/j.cell.2011.02.008 21458672 PMC3073827

[B100] Sachse S. Beshel J. (2016). The good, the bad, and the hungry: how the central brain codes odor valence to facilitate food approach in Drosophila. *Curr. Opin. Neurobiol.* 40 53–58. 10.1016/j.conb.2016.06.012 27393869 PMC5056820

[B101] Sayin S. De Backer J.-F. Siju K. P. Wosniack M. E. Lewis L. P. Frisch L.-M. (2019). A Neural Circuit Arbitrates between Persistence and Withdrawal in Hungry Drosophila. *Neuron* 104 544.e–558.e. 10.1016/j.neuron.2019.07.028 31471123 PMC6839618

[B102] Scheiner R. (2004). Activity of cGMP-Dependent Protein Kinase (PKG) Affects Sucrose Responsiveness and Habituation in Drosophila melanogaster. *Learn. Memory* 11 303–311. 10.1101/lm.71604 15169860 PMC419733

[B103] Schützler N. Girwert C. Hügli I. Mohana G. Roignant J.-Y. Ryglewski S. (2019). Tyramine action on motoneuron excitability and adaptable tyramine/octopamine ratios adjust *Drosophila* locomotion to nutritional state. *Proc. Natl. Acad. Sci. USA* 116 3805–3810. 10.1073/pnas.1813554116 30808766 PMC6397572

[B104] Seidenbecher S. E. Sanders J. I. von Philipsborn A. C. Kvitsiani D. (2020). Reward foraging task and model-based analysis reveal how fruit flies learn value of available options. *PLoS One* 15:e0239616. 10.1371/journal.pone.0239616 33007023 PMC7531776

[B105] Selcho M. Pauls D. (2019). Linking physiological processes and feeding behaviors by octopamine. *Curr. Opin. Insect Sci.* 36 125–130. 10.1016/j.cois.2019.09.002 31606580

[B106] Silva V. Palacios-Muñoz A. Okray Z. Adair K. L. Waddell S. Douglas A. E. (2021). The impact of the gut microbiome on memory and sleep in *Drosophila*. *J. Exp. Biol.* 224:jeb233619. 10.1242/jeb.233619 33376141 PMC7875489

[B107] Sitaraman D. Kramer E. F. Kahsai L. Ostrowski D. Zars T. (2017). Discrete Serotonin Systems Mediate Memory Enhancement and Escape Latencies after Unpredicted Aversive Experience in Drosophila Place Memory. *Front. Syst. Neurosci.* 11:92. 10.3389/fnsys.2017.00092 29321732 PMC5732137

[B108] Sitaraman D. LaFerriere H. (2020). Finding a place and leaving a mark in memory formation. *J. Neurogenet.* 34 21–27. 10.1080/01677063.2019.1706094 31878832 PMC7124997

[B109] Sitaraman D. Zars M. LaFerriere H. Chen Y.-C. Sable-Smith A. Kitamoto T. (2008). Serotonin is necessary for place memory in Drosophila. *Proc. Natl. Acad. Sci.* 105 5579–5584. 10.1073/pnas.0710168105 18385379 PMC2291120

[B110] Sitaraman D. Zars T. (2010). Lack of prediction for high-temperature exposures enhances Drosophila place learning. *J. Exp. Biol.* 213 4018–4022. 10.1242/jeb.050344 21075943

[B111] Soibam B. Mann M. Liu L. Tran J. Lobaina M. Kang Y. Y. (2012). Open-field arena boundary is a primary object of exploration for *Drosophila*. *Brain Behav.* 2 97–108. 10.1002/brb3.36 22574279 PMC3345355

[B112] Stafford J. W. Lynd K. M. Jung A. Y. Gordon M. D. (2012). Integration of Taste and Calorie Sensing in Drosophila. *J. Neurosci.* 32 14767–14774. 10.1523/JNEUROSCI.1887-12.2012 23077061 PMC6621435

[B113] Stam R. (2007). PTSD and stress sensitisation: A tale of brain and body Part 2: Animal models. *Neurosci. Biobehav. Rev.* 31 558–584. 10.1016/j.neubiorev.2007.01.001 17350095

[B114] Stern U. Srivastava H. Chen H.-L. Mohammad F. Claridge-Chang A. Yang C.-H. (2019). Learning a Spatial Task by Trial and Error in Drosophila. *Curr. Biol.* 29 2517.e–2525.e. 10.1016/j.cub.2019.06.045 31327716

[B115] Tatar M. Post S. Yu K. (2014). Nutrient control of Drosophila longevity. *Trends Endocrinol. Metab.* 25 509–517. 10.1016/j.tem.2014.02.006 24685228 PMC4177520

[B116] Tierney A. J. (2020). Feeding, hunger, satiety and serotonin in invertebrates. *Proc. R. Soc. B.* 287:20201386. 10.1098/rspb.2020.1386 32781950 PMC7575527

[B117] Tsao C.-H. Chen C.-C. Lin C.-H. Yang H.-Y. Lin S. (2018). Drosophila mushroom bodies integrate hunger and satiety signals to control innate food-seeking behavior. *eLife* 7:e35264. 10.7554/eLife.35264 29547121 PMC5910021

[B118] Vaziri A. Khabiri M. Genaw B. T. May C. E. Freddolino P. L. Dus M. (2020). Persistent Epigenetic Reprogramming of Sweet Taste by Diet. *Neuroscience* 2020:007773. 10.1101/2020.03.25.007773 PMC7673743 33177090

[B119] Wang G. Wang L. (2019). Recent advances in the neural regulation of feeding behavior in adult Drosophila. *J. Zhejiang Univ. Sci. B* 20 541–549. 10.1631/jzus.B1900080 31168968 PMC6587000

[B120] Wang P. Jia Y. Liu T. Jan Y.-N. Zhang W. (2020a). Visceral Mechano-sensing Neurons Control Drosophila Feeding by Using Piezo as a Sensor. *Neuron* 108 640.e–650.e. 10.1016/j.neuron.2020.08.017 32910893 PMC8386590

[B121] Wang Q.-P. Lin Y. Q. Lai M.-L. Su Z. Oyston L. J. Clark T. (2020b). PGC1α Controls Sucrose Taste Sensitization in Drosophila. *Cell Reports* 31:107480. 10.1016/j.celrep.2020.03.044 32268099

[B122] Wang Q.-P. Lin Y. Q. Zhang L. Wilson Y. A. Oyston L. J. Cotterell J. (2016). Sucralose Promotes Food Intake through NPY and a Neuronal Fasting Response. *Cell Metab.* 24 75–90. 10.1016/j.cmet.2016.06.010 27411010

[B123] Wang Q.-P. Simpson S. J. Herzog H. Neely G. G. (2017). Chronic Sucralose or L-Glucose Ingestion Does Not Suppress Food Intake. *Cell Metab.* 26 279–280. 10.1016/j.cmet.2017.07.002 28768164

[B124] Wu C.-L. Chang C.-C. Wu J.-K. Chiang M.-H. Yang C.-H. Chiang H.-C. (2018). Mushroom body glycolysis is required for olfactory memory in Drosophila. *Neurobiol. Learn. Memory* 150 13–19. 10.1016/j.nlm.2018.02.015 29477608

[B125] Yamagata N. Ichinose T. Aso Y. Plaçais P.-Y. Friedrich A. B. Sima R. J. (2015). Distinct dopamine neurons mediate reward signals for short- and long-term memories. *Proc. Natl. Acad. Sci. USA* 112 578–583. 10.1073/pnas.1421930112 25548178 PMC4299218

[B126] Yang Z. Yu Y. Zhang V. Tian Y. Qi W. Wang L. (2015). Octopamine mediates starvation-induced hyperactivity in adult *Drosophila*. *Proc. Natl. Acad. Sci.* 112 5219–5224. 10.1073/pnas.1417838112 25848004 PMC4413307

[B127] Yellman C. Tao H. He B. Hirsh J. (1997). Conserved and sexually dimorphic behavioral responses to biogenic amines in decapitated Drosophila. *Proc. Natl. Acad. Sci.* 94 4131–4136. 10.1073/pnas.94.8.4131 9108117 PMC20580

[B128] Youn H. Kirkhart C. Chia J. Scott K. (2018). A subset of octopaminergic neurons that promotes feeding initiation in Drosophila melanogaster. *PLoS One* 13:e0198362. 10.1371/journal.pone.0198362 29949586 PMC6021039

[B129] Zhang S. X. Rogulja D. Crickmore M. A. (2019). Recurrent Circuitry Sustains Drosophila Courtship Drive While Priming Itself for Satiety. *Curr. Biol.* 29 3216.e–3228.e. 10.1016/j.cub.2019.08.015 31474539 PMC6783369

[B130] Zhang Y. Liu G. Yan J. Zhang Y. Li B. Cai D. (2015). Metabolic learning and memory formation by the brain influence systemic metabolic homeostasis. *Nat. Commun.* 6:6704. 10.1038/ncomms7704 25848677 PMC4391062

