# Supplementary figures and images for: The Panopticon—Assessing the Effect of Starvation on Prolonged Fly Activity and Place Preference
Source: Front Behav Neurosci. 2021 Mar 25;15:640146. doi: 10.3389/fnbeh.2021.640146 (PMC8026880; doi:10.3389/fnbeh.2021.640146)

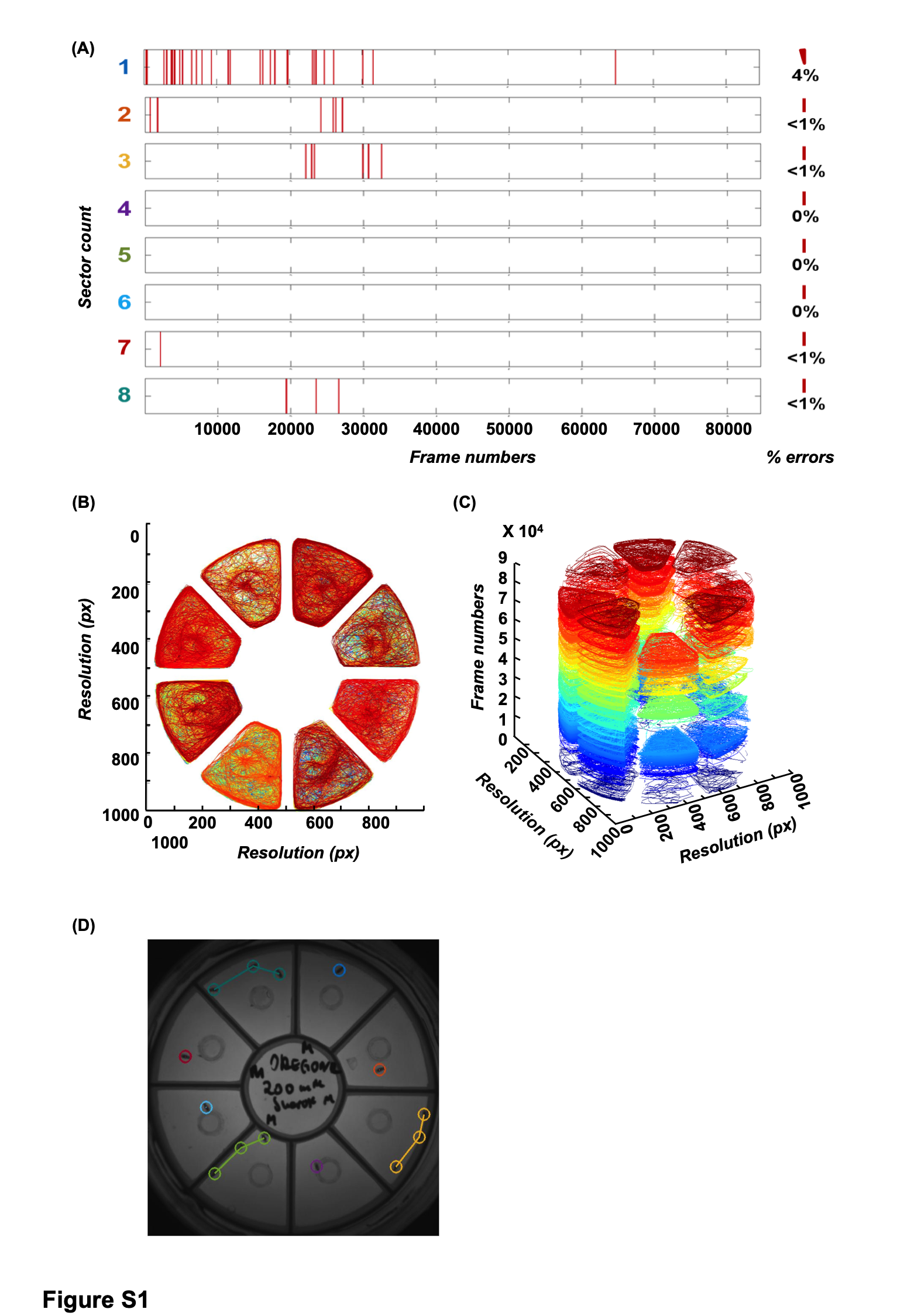

Supplement: Supplementary Figure 1 — Representative plots for multi-step quality check. (A) Temporal distribution of failed/missed detections with total error rate (%) indicated on the right panel for individual flies/sectors across 24 h. (B) Walking path traces for the entire arena divided by sectors, with (C) 3D representation, expandable on the Z-axis representing walking traces across the total duration of activity recording for 24 h. Time scales represented as frame numbers (@ 1 frame/s). Blue-yellow-red colour transition represents walking traces from 0-n frame count (across total duration of the experiment). (D) To visually identify long-distance movements associated with potentially false-positive detections, corresponding images for top ten frames with flies covering the longest distances can be extracted (example image for Sector 8). Such images include up to 2 consecutive long distance moves as indicated by individually coloured walking trace lines and the fly itself encircled with the respective colour. Apart from the queried sector, all the other sectors also indicate distances moves in corresponding frames. [file Image_1.TIFF]
